# Do Psychosocial Resources Mitigate the Impact of Adverse Childhood Experiences in Oral Health‐Related Quality of Life Among Adolescents

**DOI:** 10.1111/ipd.70081

**Published:** 2026-03-23

**Authors:** Thaís Gioda Noronha‐Ramos, Jessica Klöckner Knorst, Thiago Machado Ardenghi, Bruno Emmanuelli, Bruna Brondani, Fernanda Tomazoni

**Affiliations:** ^1^ Post Graduate Program in Dental Sciences Federal University of Santa Maria Santa Maria Brazil; ^2^ Department of Stomatology, School of Dentistry Federal University of Santa Maria Santa Maria Brazil

**Keywords:** adolescent, adverse childhood experience, oral health, psychosocial factors, quality of life

## Abstract

**Background:**

Adverse childhood experiences (ACEs) are associated with poorer general and oral health outcomes, but the psychosocial mechanisms underlying these associations remain unclear.

**Aim:**

The aim of this study is to assess whether perceived social support and sense of coherence (SOC) moderate the association between ACEs and oral health‐related quality of life (OHRQoL) among adolescents.

**Design:**

A cross‐sectional study nested within a cohort of 406 adolescents aged 14–18 years from southern Brazil. OHRQoL was measured using the Child Perceptions Questionnaire (CPQ11‐14), ACEs by the Adverse Childhood Experiences–International Questionnaire (ACE‐IQ), perceived social support by a proxy question, and SOC by the 13‐item Sense of Coherence Scale (SOC‐13). Sociodemographic and behavioral variables were included as covariates. Poisson regression was conducted.

**Results:**

Adolescents exposed to ACEs and reporting low perceived social support had CPQ11‐14 scores 1.48 (95% CI 1.26–1.74) times higher than peers without ACEs and with high support. Those with ACEs and low SOC had scores 2.5 times higher (95% CI 2.22–2.80). Adolescents with ACEs but higher perceived social support or SOC levels also exhibited higher CPQ11‐14 scores, although with smaller impacts.

**Conclusion:**

These findings emphasize that psychosocial factors, such as perceived social support and SOC, may buffer the impact of ACEs on OHRQoL in adolescents.

## Why This Paper Is Important to Pediatric Dentists

- Adverse childhood experiences (ACEs) negatively influence adolescents' oral health‐related quality of life, highlighting the relevance of psychosocial factors in dental care.
- Perceived social support and sense of coherence (SOC) act as protective factors that can buffer the impact of ACEs on oral health outcomes.
- Understanding these psychosocial mechanisms can help pediatric dentists adopt a more holistic approach, integrating emotional and social dimensions into preventive and therapeutic strategies for adolescents.

## 1 Introduction

Adverse childhood experiences (ACEs) refer to various forms of abuse and household dysfunction experienced from birth to 18 years of age, including physical, emotional, and sexual abuse, as well as exposure to domestic violence, substance misuse, parental mental illness, and parental separation or incarceration [1, 2]. A growing body of evidence has linked ACEs to a range of adverse health outcomes, such as lower educational attainment, engagement in health‐risk behaviors, and increased incidence of chronic conditions, including diabetes, obesity, cardiovascular and respiratory diseases, liver disorders, and oral health problems [2, 3]. These associations may result from alterations in physiological development and the persistence of harmful behaviors triggered by early‐life adversity [2].

Evidence from the literature supports an association between ACEs and poorer oral health outcomes, including a higher number of missing teeth, fewer remaining teeth, increased self‐reported dental caries, greater dental fear and anxiety, and lower oral health‐related quality of life (OHRQoL) [4]. Moreover, studies have demonstrated a dose–response relationship, indicating that a greater number of adverse experiences during childhood is associated with more severe health consequences [1, 5]. Thus, although the negative association between ACEs and various oral health outcomes is well‐established, further research is needed to identify psychosocial factors that may modify or buffer these associations, which may inform strategies to reduce or mitigate the long‐term impacts of early adversity.

Previous results have identified potential moderators that may buffer the impact of ACEs on general health, including social support and coping strategies [6]. Social support refers to the perception or experience of being cared for, valued, and integrated into a social network that offers emotional, informational, or practical assistance in times of need [7]. Sense of coherence (SOC), a central concept of the salutogenic model, reflects an individual's capacity to perceive life as comprehensible, manageable, and meaningful, enabling more effective responses to stress and adversity [8]. These psychosocial factors have also been shown to moderate the effects of disease burden on well‐being and OHRQoL [9], a multidimensional construct describing the extent to which oral health conditions affect daily functioning and overall quality of life [10]. However, to the best of our knowledge, no study has yet examined whether social support and SOC specifically moderate the association between ACEs and OHRQoL in adolescents, representing a gap in the literature that the present study aims to address.

Understanding how psychosocial factors such as social support and SOC can buffer the association between ACEs and subjective oral health is essential to reducing long‐term negative outcomes across the lifespan. Within the life course epidemiological framework—particularly the risk accumulation model, which considers how both adverse and protective exposures accumulate over time and influence health depending on their number, duration, and severity—it is crucial to identify ACEs as early as possible [11]. Therefore, the present study aimed to evaluated the moderating effect of perceived social support and SOC on the relationship between ACEs and OHRQoL among adolescents. We hypothesized that both psychosocial factors would attenuate the strength of this association.

## 2 Materials and Methods

This study was conducted in accordance with the Strengthening the Reporting of Observational Studies in Epidemiology (STROBE) guidelines [12]. Ethical approval was granted by the Research Ethics Committee of the Federal University of Santa Maria (CAAE: 63937422.0.0000.5346), and authorization was obtained from both municipal and regional education authorities. Written informed consent was obtained from the legal guardians of all participants, and written assent was provided by the adolescents themselves.

### 2.1 Study Design and Participants

This cross‐sectional study included participants from a representative cohort initiated in 2010 in Santa Maria, southern Brazil, as previously described [13, 14, 15]. The present analysis is based solely on data from the most recent follow‐up, conducted in 2023, involving adolescents aged 14 to 18 years.

A post hoc power analysis was conducted to verify whether the available sample was sufficient to detect the interaction effects of interest. Based on a significance level of 0.05, a statistical power of 80%, and a minimum effect size of 0.3 as proposed by Cohen (1988) [16], the analysis confirmed that the final sample of 406 participants provided adequate power for the proposed analyses.

### 2.2 Data Collection, Variables and Instruments

All variables were assessed using validated instruments. Data collection was conducted by trained dental professionals through face‐to‐face interviews using a structured questionnaire. The selection of items and indices was guided by a comprehensive literature review and aligned with a robust theoretical framework.

### 2.3 Adverse Childhood Experiences

ACEs were assessed using items from the Family Environment Section of the Brazilian version of the Adverse Childhood Experiences–International Questionnaire (ACE‐IQ) [17]. This instrument was self‐administered by adolescents and referred to events occurring during the first 18 years of life. The questions addressed household dysfunction and included the following items: “Did you live with anyone who was a problem drinker or alcoholic or who used street drugs?”, “Was a household member depressed or mentally ill, or did a household member attempt suicide?”, “Did a household member go to prison?”, “Were your parents ever separated or divorced?”, and “Did your mother, father, or guardian die?”. Response options were “Yes,” “No,” and “Don't know or don't want to answer” (considered missing data). For analytical purposes, ACEs were categorized as present or absent of at least one ACE.

### 2.4 Oral Health‐Related Quality of Life

To assess OHRQoL, the Brazilian short version of the Child Perceptions Questionnaire for ages 11–14 (CPQ11‐14) was used, as this instrument has been consistently applied across age ranges beyond those originally specified by the questionnaire [18, 19]. This instrument consists of 16 items that evaluate the frequency of events experienced over the past three months, being divided into four domains: oral symptoms, functional limitations, social well‐being, and emotional well‐being. Each item is scored on a five‐point Likert scale with response options ranging from “never” = 0 to “every day or almost every day” = 4. The total score is obtained by summing the responses to all items, yielding a possible range from 0 to 64. Higher scores indicate a greater negative impact on OHRQoL [19].

### 2.5 Perceived Social Support

Perceived social support was assessed using the following question, considered a proxy for the support network: “If something unfortunate happened to you, would someone help you in this situation?” with response options: yes, or not [7]. For the purposes of data analysis, the presence or absence of perceived social support was considered.

### 2.6 Sense of Coherence

SOC was measured using the short version of the 13‐item Sense of Coherence Scale (SOC‐13) validated for use in Brazil [20, 21]. Responses are given on a 5‐point Likert scale, with response options varying depending on the item and are coded from 1 to 5. The final SOC score is calculated by summing all item scores, yielding a total score ranging from 13 to 65, with higher scores indicating a stronger SOC. For data analysis, SOC‐13 scores were dichotomized at the median into low SOC (≤ 48) and high SOC (> 48).

### 2.7 Covariates

Demographics, socioeconomic, and behavioral variables were used as adjustment factors. Skin color was measured according to the classification proposed by the Brazilian Institute of Geography and Statistics (IBGE), which is widely used in population‐based surveys in Brazil. For data analysis, responses were dichotomized into “white” and “black‐brown.” Monthly household income was collected in Brazilian currency and subsequently dichotomized based on the Brazilian Minimum Wage (BMW), into ≤ 1 BMW or > 1 BMW (1 BMW is approximately equivalent to US$240.00). Regular use of services was assessed through the following question: “What was the reason for your last consultation?” and was dichotomized into routine and non‐routine reasons.

### 2.8 Statistical Analysis

Data analysis was performed using Stata version 17 (StataCorp, College Station, TX). A descriptive analysis of the sample was performed. Both unadjusted and adjusted Poisson regression analyses with robust variance were conducted to examine the moderating role of perceived social support and SOC in the association between ACEs and OHRQoL (CPQ11‐14). A moderation effect is observed when the strength or direction of the relationship between two variables varies as a function of a third variable—the moderator [22]. Interaction terms were tested on a multiplicative scale, in line with previous studies, to assess potential effect modification [9, 23]. The interactions evaluated included ACEs (no vs. yes) × Perceived Social Support (high vs. low) and ACEs (no vs. yes) × SOC (high vs. low). To further explore significant interactions, simple slope analyses were conducted to estimate the conditional effect of ACEs on OHRQoL at different levels of the moderator variables. The results are reported as rate ratio (RR) with 95% confidence intervals (CI). A *p*‐value < 0.05 was considered statistically significant.

## 3 Results

A total of 406 adolescents were evaluated. The sample was balanced by sex, with a mean age of 15.5 years (SD 1.3). Regarding self‐reported skin color, 67.3% identified as white. Additionally, 23.4% of the participants reported a household income below that of a BMW. The prevalence of ACEs was 66.7%. Concerning psychosocial variables, 7.7% of the adolescents reported low perceived social support, and 57.5% presented with low SOC. The mean score on the CPQ11‐14 was 10.9 (SD 8.1) (Table 1).

**TABLE 1:** General characteristics of the sample, Santa Maria, RS, Brazil, (*n* = 406).

Variables	*N*	%
*Potential confunders*		
Sex		
Girls	209	51.5
Boys	197	48.5
Age		
14	75	18.9
15	83	20.9
16	91	22.9
17	148	37.3
Skin color		
White	66	67.3
Black or brown	32	32.7
Household income in BMW		
≥ 1 BMW	279	76.6
< 1 BMW	85	23.4
Use of services		
Routine	229	61.2
No‐routine	145	38.8
*Interactions variables*		
ACE		
No	128	33.3
Yes	257	66.7
Perceived social support		
High	374	92.3
Low	31	7.7
Sense of coherence		
High	171	42.3
Low	233	57.7
*Outcome*	Mean	SD
OHRQoL (CPQ11‐14)	10.9	8.1

Abbreviations: ACE, adverse childhood experiences; BMW, Brazilian minimum wage; CPQ, child perception questionnaire; OHRQoL, oral health‐related quality of life; SD, standard deviation.

Table 2 presents the predictive margins and unadjusted analyses assessing the associations between ACEs, perceived social support, SOC, and their interactions with overall CPQ11‐14 scores. Exposure to ACEs, low perceived social support, and low SOC were associated with higher CPQ11‐14 scores, reflecting poorer OHRQoL. Regarding interaction effects, among adolescents that experienced ACEs, the mean CPQ11‐14 score was 11.6 (SE 0.2) in those with high perceived social support and 12.8 (SE 0.8) in those with low perceived social support. Similarly, the mean CPQ11‐14 score was 7.7 (SE 0.2) in adolescents with high SOC and 14.2 (SE 0.8) in those with low SOC.

**TABLE 2:** Predictive margins and unadjusted analysis of ACE, perceived social support, sense of coherence and interactions on overall CPQ11‐14 scores.

Variables	*N*	OHRQoL (CPQ11‐14)		
		Mean (SE)	RR (95% CI)	*p*‐value
ACE				
No	128	8.6 (0.3)	1 (reference)	
Yes	257	11.7 (0.4)	1.35 (1.26–1.45)	< 0.001
Perceived social support				
High	374	10.7 (0.4)	1 (reference)	
Low	31	12.1 (0.6)	1.12 (1.01–1.24)	< 0.05
Sense of coherence				
High	171	7.0 (0.2)	1 (reference)	
Low	233	13.6 (0.4)	1.94 (1.81–2.07)	< 0.001
*Interaction variables*				
ACE × Perceived Social Support				
Not‐High	117	8.6 (0.2)	1 (reference)	
Not‐Low	11	8.6 (0.8)	0.99 (0.80–1.23)	0.976
Yes‐High	240	11.6 (0.2)	1.34 (1.25–1.44)	< 0.001
Yes—Low	17	12.8 (0.8)	1.48 (1.28–1.72)	< 0.001
ACE × Sense of Coherence				
Not‐High	71	5.8 (0.2)	1 (reference)	
Not‐Low	57	12.0 (0.4)	2.05 (1.81–2.31)	< 0.001
Yes‐High	97	7.7 (0.2)	1.30 (1.16–1.47)	< 0.001
Yes‐Low	159	14.2 (0.2)	2.41 (2.17–2.68)	< 0.001

Abbreviations: ACE, adverse childhood experiences; CI, confidence interval; CPQ, child perception questionnaire; OHRQoL, oral health‐related quality of life; RR, rate ratio; SE, standard error.

Table 3 displays the adjusted analysis between the interaction variables and overall CPQ11‐14 scores. Adolescents who had experienced ACEs and reported low perceived social support had CPQ11‐14 scores 1.48 times higher (RR 1.48; 95% CI 1.26–1.74) than those without ACEs and with high perceived social support. Similarly, individuals exposed to ACEs and with low SOC had approximately 2.5 times higher scores (RR 2.50; 95% CI 2.22–2.80) than their counterparts. Although adolescents with ACEs combined with either high perceived social support or high SOC also exhibited higher CPQ11‐14 scores compared to those without ACEs, the magnitude of these associations was notably smaller—particularly among those with high SOC.

**TABLE 3:** Adjusted analysis of the interaction of ACE with Perceived Social Support and Sense of Coherence on overall CPQ11‐14 scores.

*Interaction variables*		OHRQoL (CPQ11‐14)	
	*N*	RR (95% CI)	*p*‐value
ACE × Perceived Social Support			
Not—High	117	1 (reference)	
Not—Low	11	1.08 (0.87–1.34)	0.458
Yes‐High	240	1.34 (1.24–1.45)	< 0.001
Yes‐Low	17	1.48 (1.26–1.74)	< 0.001
ACE × Sense of Coherence			
Not‐High	71	1 (reference)	
Not‐Low	57	2.16 (1.89–2.46)	< 0.001
Yes‐High	97	1.36 (1.19–1.56)	< 0.001
Yes‐Low	159	2.50 (2.22–2.80)	< 0.001

Abbreviations: ACE, adverse childhood experiences; CI, confidence interval; CPQ, child perception questionnaire; OHRQoL, oral health‐related quality of life; RR, rate ratio.

## 4 Discussion

This study investigated the moderating role of perceived social support and SOC in the association between ACEs and OHRQoL in adolescents. The findings revealed that both perceived social support and SOC were associated with attenuation of ACEs on OHRQoL. Among adolescents exposed to ACEs, those with higher levels of perceived social support and SOC experienced less impairment in OHRQoL compared to those with lower levels, thereby supporting the study's conceptual hypothesis.

This finding supports the well‐established notion that social support serves as a protective factor in the context of adversity. Other studies that evaluated childhood maltreatment and different mental and physical health outcomes also showed that social support was able to mitigate these relationships [6, 24]. Social support may buffer the negative effects of early life stress on health outcomes by enhancing coping mechanisms, reducing psychological distress, and promoting health‐related behavior [25, 26]. In adolescents, perceived support from family, peers, or significant adults has been associated with better mental and physical health outcomes, including [27, 28]. The interaction observed in our study suggests that even in the presence of early adversity, higher levels of perceived social support were associated with less negative impact on OHRQoL, underscoring its relevance as a target for public health interventions.

Similarly, adolescents who experienced ACEs and exhibited low SOC had higher CPQ11‐14 scores than those without ACEs and with high SOC. This association highlights the potential role of SOC as a psychosocial resource that may buffer the adverse association between early life stress and subjective oral health outcomes. SOC reflects an individual's capacity to perceive life as comprehensible, manageable, and meaningful, and has been shown to be inversely associated with perceived stress and poor health outcomes in both general and adolescent populations [8, 20]. In the context of oral health, individuals with stronger SOC are more likely to engage in health‐promoting behaviors and to interpret stressful situations in ways that do not compromise their well‐being [29, 30]. Therefore, our findings are consistent with the hypothesis that low SOC may be associated with amplification of the psychological and behavioral consequences of ACEs, contributing to poorer OHRQoL in adolescents.

Although adolescents with ACEs and either high perceived social support or high SOC still exhibited higher CPQ11‐14 scores compared to those without ACEs, the magnitude of these associations was smaller, particularly among those with high SOC. This reinforces that exposure to ACEs is generally associated with poorer OHRQoL, regardless of protective factors. However, among those who have experienced adversity, higher levels of perceived social support and especially a strong SOC were associated with attenuation of negative impact. The stronger association observed for SOC compared to perceived social support may reflect its broader and more internalized nature, encompassing not only external support but also individual coping capacity and resilience [8, 20]. As SOC is often shaped through supportive environments over time, it may integrate both the presence of social resources and the personal ability to make sense of and manage stressors, which could explain its more pronounced association with OHRQoL in this context. However, this interpretation remains speculative and should be considered with caution. The difference in effect magnitude may also reflect methodological factors, including the distinct measurement properties of each instrument, namely, the multidimensional and psychometrically robust nature of the SOC‐13 scale compared to the single‐item measure of perceived social support, as well as the analytical decision to dichotomize both variables.

This study has some limitations that should be acknowledged. Although it is nested within a cohort, all data used are cross‐sectional in nature. Nevertheless, we emphasize that this is a relatively unexplored topic in the dental literature, and our study represents an important contribution to this growing body of research. There is still much to investigate regarding this subject. Additionally, the assessment of ACEs was restricted to household dysfunction items from the ACE‐IQ, excluding domains related to direct abuse and neglect. This decision was made to minimize psychological distress in a school‐based setting and to ensure ethical compliance; however, it likely underestimates the true prevalence of ACE exposure. Consequently, the associations reported in this study should be interpreted as conservative estimates, and the inclusion of additional ACE domains in future research could reveal stronger associations or different moderating patterns. However, it is noteworthy that this limitation did not preclude the identification of significant associations in our analysis, highlighting the evident protective effect of social support in the relationship between ACEs and OHRQoL.

Furthermore, perceived social support was assessed using a single‐item measure, which may not fully capture the multidimensional nature of this construct. Any misclassification arising from this measure is likely to be non‐differential, that is, not systematically related to ACE exposure or OHRQoL, which would tend to attenuate rather than inflate the observed associations, suggesting that the true protective effect of perceived social support may be even stronger than reported here. Finally, both ACEs and SOC were dichotomized for analytical purposes, which may have reduced variability and obscured potential dose–response relationships. However, this approach is consistent with how these variables have been operationalized in previous studies in the oral health literature [1, 4, 5, 15].

Our findings also present several strengths. This study supports previous research by reinforcing the association between ACEs and OHRQoL. It is noteworthy that OHRQoL was assessed using a validated questionnaire, whereas some studies evaluating this association have measured the outcome using a single‐item proxy [4]. Additionally, we identified potential moderating variables that were associated with attenuation of this relationship, offering a more optimistic perspective given the non‐negligible prevalence of ACEs in the sample studied. These results underscore the importance of integrating psychosocial dimensions into oral health promotion strategies, particularly for vulnerable populations. Furthermore, future longitudinal research is warranted to explore the potential pathways linking ACEs to OHRQoL. Future interventions should also consider strengthening social support networks and resilience‐related attributes to mitigate the long‐term consequences of early life adversity on oral health outcomes.

In conclusion, the findings of this study underscore the importance of integrating psychosocial screening into pediatric dental practice, particularly for adolescents with a history of adverse childhood experiences. Perceived social support and sense of coherence emerged as significant moderators of the association between ACEs and OHRQoL, highlighting the potential value of strengthening these psychosocial resources as part of oral health promotion strategies for vulnerable populations. Future longitudinal research is warranted to examine the pathways linking ACEs to OHRQoL and to test interventions targeting social support networks and resilience‐related attributes.

## Author Contributions

T.G.N.‐R. and J.K.K. – Investigation, Data curation, Formal analysis, Conceptualization, Writing – original draft, Review ans editing; T.M.A. – Writing – review and editing, Investigation, Funding acquisition, Project administration; B.E. – Writing – review and editing, Investigation, Funding acquisition, Project administration; B.B. – Writing – review and editing, Investigation; F.T. – Formal analysis, Conceptualization, Funding acquisition, Review and editing.

## Funding

This work was supported by Coordenação de Aperfeiçoamento de Pessoal de Nível Superior, 001. Fundação de Amparo à Pesquisa do Estado do Rio Grande do Sul, 21/2551‐0002006‐7.

## Ethics Statement

The study is in accordance with recognized ethical standards, including the Declaration of Helsinki. Ethical approval was obtained from the Research Ethics Committee of the Federal University of Santa Maria (CAAE: 63937422.0.0000.5346). Written consent was provided by guardians and assent by adolescents.

## Conflicts of Interest

The authors declare no conflicts of interest.

## Data Availability

The data that support the findings of this study are available on request from the corresponding author. The data are not publicly available due to privacy or ethical restrictions.
